# Tophaceous Gout of the Middle Ear

**DOI:** 10.5334/jbsr.3421

**Published:** 2024-02-05

**Authors:** Joren Waumans, Christian Desloovere, Johannes Devos

**Affiliations:** Department of Radiology, University Hospitals Leuven, Herestraat 49, Leuven, 3000, Belgium, Email: joren.waumans@uzleuven.be; Department of Otorhinolaryngology, Head and Neck Surgery, University Hospitals Leuven, Herestraat 49, Leuven, 3000, Belgium, Email: christian.desloovere@uzleuven.be; Department of Radiology, University Hospitals Leuven, Herestraat 49, Leuven, 3000, Belgium, Email: johannes.1.devos@uzleuven.be

**Keywords:** Gout, middle ear, tomography, ear ossicles, conductive hearing loss

## Abstract

Tophaceous gout can rarely present in the middle ear as a mass-like lesion, causing conductive hearing loss. Noncontrast high-resolution computed tomography (HRCT) of the temporal bone plays a significant role in the diagnosis. Awareness of this condition among radiologists is important since it presents a distinctive appearance on HRCT. We present a case of tophaceous gout of the middle ear diagnosed with photon-counting computed tomography (PCCT).

*Teaching point:* The presence of a partially calcified mass with a semolina-like appearance within the middle ear is highly suggestive of tophaceous gout, even in the presence of normal serum uric acid levels.

## Introduction

Gout (monosodium urate crystal deposition disease) is an inflammatory arthropathy and ranks among the primary causes of monoarthritis, primarily affecting peripheral joints such as the great toe or the knee [1]. Manifestations in the head and neck region have rarely been reported, affecting the sternoclavicular, temporomandibular, and cricoarytenoid joints, as well as the larynx, nose, pinna, and middle ear [1–5].

## Case History

A 75-year-old female patient presented to the Department of Otorhinolaryngology due to an episode of instability. A routine examination revealed progressive conductive hearing loss on the right side. A photon-counting computed tomography (PCCT) was performed to investigate the etiology of this hearing loss. Notably, there were no typical gout symptoms in other joints at the time of the clinical assessment, and serum uric acid levels were within the normal range.

**Figure 1:**
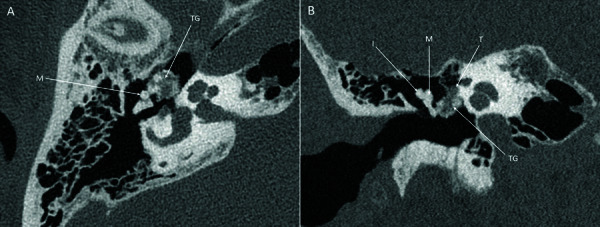
PCCT images (SIEMENS NAEOTOM Alpha) of the right temporal bone. (A) The axial image shows contact of the tophaceous gout (TG) with the malleus (M). (B) The coronal image shows contact with the tympanic segment of the facial nerve (T) and the malleus (M). The incus (I) is not affected.

PCCT of the temporal bone revealed a partially calcified mass measuring 7 × 6 × 4 mm located anteriorly in the epitympanum with some extension into the mesotympanum. The density of the mass was heterogeneous with calcifications intermixed with foci of soft tissue density. The mass was medially in contact with the tympanic segment of the facial nerve canal and laterally with the malleus (Figure 1). Based on the imaging findings, tophaceous gout of the middle ear was suspected.

**Figure 2:**
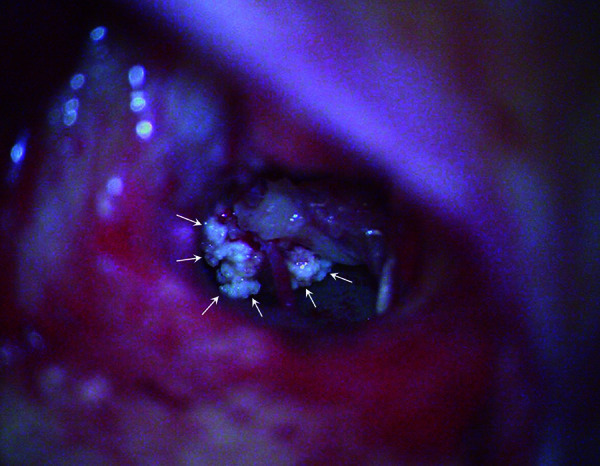
Intraoperative view of the middle ear, where the tophaceous gout is seen as a white and crystalline mass (arrows).

**Figure 3:**
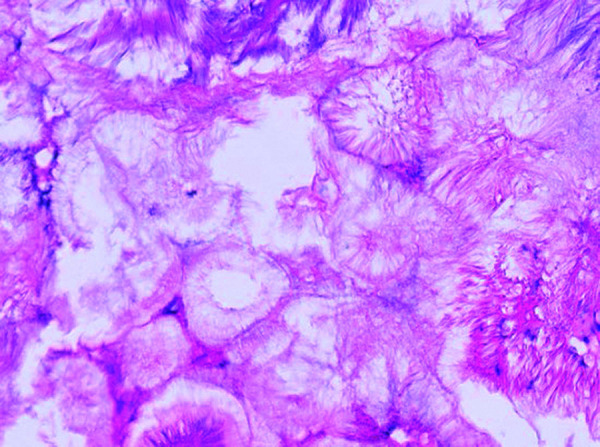
A hematoxylin and eosin-stained slide (scale 50 µm) shows the presence of empty needle-like structures with a feathery appearance, characteristic of gout tophi (Courtesy of A. Vanstapel MD, PhD).

Given the probable causative relation of the mass with the conductive hearing loss, surgical intervention involving tympanoplasty with resection of the mass was performed. Intraoperatively, the mass was white and relatively soft with a crystalline appearance (Figure 2). Ossicular chain continuity was reestablished through incus remodeling. Pathological examination of the mass confirmed the radiological suspicion of a gout tophus (Figure 3).

Follow-up at 2 and 6 months post-surgery revealed only a marginal hearing improvement on audiometry alongside favorable healing on otomicroscopy. Subsequent CT examination showed suboptimal contact between the remodeled incus and the stapes. A millimetric calcified mass was also noted anteriorly in the middle ear. A revision tympanoplasty was performed, revealing a residual or recurrent gout tophus, which was resected. Additionally, a total ossicular replacement prosthesis (TORP) was implanted for a fracture of the anterior crus of the stapes to improve hearing.

## Discussion

Gout is generally associated with elevated serum uric acid levels [1]. Notably, in this case, as well as in most reported cases of tophaceous gout in the middle ear, patients exhibited normal serum uric acid levels. Furthermore, no cases indicated previous gout manifestations in other joints [1–4].

The potential recurrence of tophaceous gout within 6 months after the initial surgical intervention could be a remarkable aspect of this case. To our knowledge, the recurrence of gout tophi in the middle ear has not been documented; therefore, we must keep in mind that there could have been an incomplete resection. Nevertheless, it might be prudent to perform routine follow-up imaging 3 to 6 months after surgery for this indication.

Diagnosing tophaceous gout affecting the middle ear demands a comprehensive approach that involves audiometry, imaging, and intraoperative correlation with macroscopic inspection of the mass. When there is a discrepancy between the imaging and intraoperative findings, histopathology can ultimately confirm or exclude the diagnosis by evaluating for the presence of monosodium urate crystals [1–4].

Audiometry serves to quantify the extent of conductive hearing loss. High-resolution computed tomography (HRCT) generally depicts a heterogeneous partially calcified mass within the middle ear, with a distinct radiological appearance as seen in previously reported cases [1–4]. A semolina-like mass has been used as a descriptive term. The introduction of PCCT may enhance diagnostic precision by improving spatial resolution [6].

The most common considerations on CT include osteoma, tympanosclerosis, and cholesteatoma. Osteomas typically present as more compact lesions with a higher and homogeneous density. Tympanosclerosis also presents as focal calcified densities in the middle ear but tends to be less distinctly delineated compared to osteomas. Cholesteatoma, on the other hand, seldom contains internal calcifications and often presents with more pronounced erosions of the adjacent bone [1–4].

Surgical resection of the gout tophus is the treatment of choice. Repair of the ossicular chain continuity is frequently needed using ossicular chain reconstruction techniques such as the interposition of grafts or prostheses [1–4].

## Conclusion

Tophaceous gout of the middle ear is a rare presentation of classical gout, often manifesting in the presence of normal uric acid serum levels. A gout tophus adjacent to the ossicular chain can cause conductive hearing loss. The characteristic imaging presentation is a semolina-like mass in the middle ear on HRCT of the temporal bone. The treatment of choice is surgical resection with ossicular chain reconstruction. In this case report, the potential for gout tophus recurrence in the middle ear is recognized, thereby suggesting that imaging should be considered in the follow-up of individuals affected by this condition.
